# Comparative Evidence-Based Model Choice: A Sketch of a Theory

**DOI:** 10.3390/e28010013

**Published:** 2025-12-23

**Authors:** Prasanta S. Bandyopadhyay, Samidha Shetty, Gordon Brittan

**Affiliations:** 1 Department of History and Philosophy, Montana State University, Bozeman, MT 59717, USA; psb@montana.edu (P.S.B.); gbrittan17@gmail.com (G.B.J.); 2 Department of Mathematical Sciences, Montana State University, Bozeman, MT 59717, USA

**Keywords:** explanation, prediction, BIC, AIC, desiderata for evidence function, Kyburg’s non-comparative interval-decision theory, interval-based weak dominance principle

## Abstract

An extensive literature on decision theory has been developed by both subjective Bayesians and Neyman–Pearson (NP) theorists, with more recent contributions to it from evidential decision theorists. The last-mentioned, however, have often been framed from a Bayesian perspective and therefore retain a subjectivist orientation. By contrast, we advance a comparative evidence-based model choice (CEMC) account of epistemic utility, which is explicitly non-subjective. On this account, competing models are assessed by the degree to which they are supported by the data and relevant background information, and evaluated comparatively in terms of their relative distances. CEMC thus provides a philosophical framework for inference that integrates the complementary epistemic goals of prediction and explanation. Our approach proceeds in two stages. First, we articulate a framework for non-subjective, non-NP-style, comparative, evidence-based model choice grounded in epistemic utility. Second, we identify statistical tools appropriate for measuring epistemic utility within this framework. We then contrast CEMC with non-comparative evidential decision-theoretic approaches, such as interval-based probability, pioneered by Henry Kyburg, which do not necessarily share the dual aims of explanation and prediction. We conclude by considering the interrelations between prediction, explanation, and model selection criteria, and by showing how these are closely connected with the central commitments of CEMC.

## 1. Statement of the Theory

A great deal of excellent work has already been done on Bayesian decision theory [1,2,3,4,5] and the Neyman–Pearson decision theory [6,7]. However, there is not so much on non-Bayesian evidence-based decision theory in the philosophy of science (except such as [8,9]). We will focus on this under-appreciated area of research. We propose a new form of comparative evidence-based model choice (CEMC), an epistemic utility framework in which two or more competing models are assessed based on how well each is supported by data and background information, relative to the others, in terms of their mutual distances. The relative distances between them are calculated assuming that there is a putatively “true” model. CEMC is a philosophical framework for assessing inferences based on multiple distinct but complementary goals. Common inferential goals are to (i) make predictions and (ii) provide explanations of the causal relationships between variables in the data-generating mechanisms, among others. To expedite each goal satisfactorily, investigators are often required to do one or the other or all of them together. For example, estimate a parameter inside a model, or control variables to assess their causal impact or lack thereof on other variable(s), or measure the effect size to estimate whether changing one value of a variable influences another variable’s values. Though each goal is important, they are often related to one another, and consequently, addressing one brings the other into discussion. Together with this consideration, and for the sake of brevity, we focus primarily on the CEMC framework’s goals of prediction and explanation. This need not suggest that the goals to which we will not devote time are less important. Depending on the objective, this framework adopts and adjusts statistical tools accordingly. The specific tools used are the evidence functions investigators choose to address each goal. An epistemic utility theory is usually understood as a formal account that assigns a numerical value to an agent’s degree of belief. In contrast to the conventional account, we first propose a framework for evidence-based model choice based on an epistemic utility theory that does not incorporate belief probabilities, and then provide the statistical tools used to measure epistemic utility.

**Figure 1:**
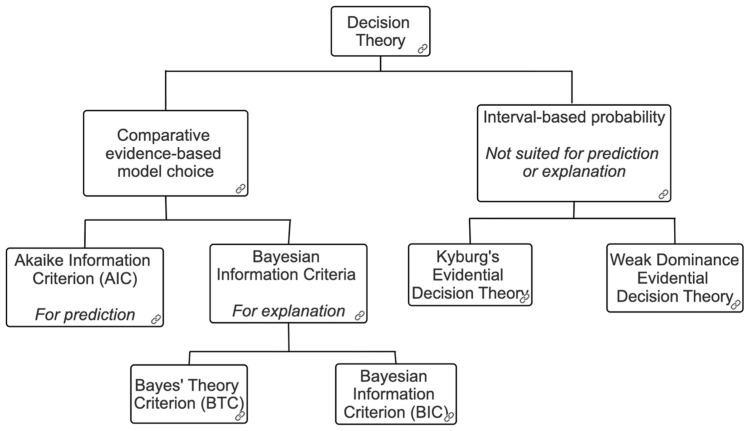
Decision theory chart.

The paper is divided into four sections. We first introduce CEMC, two primary goals of statistical models (to explain and to predict), and elaborate two types of statistical models that enable satisfaction of these goals (Section 1). We will find that both models meet distinct goals of CEMC yet choose the same hypothesis as the best. We then revisit CEMC regarding the use of divergent model selection criteria and provide an explanation of the actual data analysis using these criteria (Section 2). Though it is not surprising that different model selection criteria choose the same hypothesis as the best, it motivates us to search for a case where distinct models serve a specific goal and do not necessarily recommend the same hypothesis as the best among competing hypotheses. Here, we will broach Kepler’s search for planetary orbits, where the goal of explanation, in which a search for the causal explanation for the movements of the planets, is shown to be distinct from the goal to predict their future orbital paths. We will revisit two goals posited by CEMC: (i) to explain and (ii) to predict. We will demonstrate that varieties of Bayesian model selection criteria alone serve both goals satisfactorily (Section 3). We will then bring in another batch of evidential accounts of decision theory to show how non-comparative, non-subjective evidential decision theories work without necessarily sharing the goal of explaining or predicting events/phenomena. The latter type of decision theory is interval-based probability pioneered by Henry Kyburg’s influential work (Section 4). Finally, we conclude with some general comments about prediction, explanation, and model selection criteria, and how they are closely connected with CEMC on the other. We provide a diagram (see Figure 1) below where the caption “decision theory” represents a taxonomy regarding the evidence-based, non-NP, non-subjective decision theory to help the reader navigate the theory.

## 2. Introduction to the CEMC

Model selection criteria use what we called “statistical tools,” evidence measures, to achieve two goals. To achieve these goals, investigators are interested in knowing whether the evidence function selected to enable reaching a goal obeys some or all of the desiderata commonly held to evaluate such functions [10,11,12,13].

### 2.1. Desiderata for an Evidence Function

(a) Evidence should be a data-based estimate of the relative distance between two models and a data-generating mechanism.
(b) Evidence should be a continuous function of data. This is tantamount to saying that there is no threshold that must be passed before something will be regarded as evidence.
(c) The reliability of evidential statements should be quantifiable.
(d) Evidence should be public and not private or personal.
(e) Evidence should be portable, i.e., it should be transferable from one person to another.
(f) Evidence should be accumulable in some manner. If two data sets relate to the same pair of models, then the evidence should be combined regarding the models in question. Any evidence gathered should bear on any future inferences regarding the models in question.
(g) Evidence should not depend on the personal idiosyncrasies of model formulation. We mean by this that evidence function must be both scale- and transformation-invariant.
(h) Model consistency is important. We need to distinguish two types of consistency as one is conceptually different from the other. They are (i) the population parameter consistency and (ii) the goal of minimizing the prediction error rate. Population parameter consistency stands for the fact that M + W → 0 as *n* →∞. M is the probability that the generating process will produce data (of a certain sample size n) that leads to strong but misleading evidence. W is the probability that the generating process will produce data (of a certain sample size n) that leads to weak evidence—that is, evidence that does not strongly indicate one model or the other. M and W error probabilities are roughly comparable to Type I and Type II errors. However, they are better than Type I and Type II errors as they both go to 0 as sample size increases. It means that evidence for the true model/parameter is maximized at the true value only if the true model is in the model set or is the best projection into the model set if it is not. In other words, it ensures that, with enough data, the estimator will provide a reliable and accurate estimate of the population estimator. Another way to consider consistency is to focus on the goal for which an evidential measure is devised. The goal minimizing the prediction error rate is the other way of satisfying the consistency desideratum; a measure serves the goal both reliably and accurately if it minimizes the prediction error rate of a model. It means that the Akaikean Information Criterion (AIC) [14] selects models that have good approximations, even if they are *not* necessarily the true model.

We can construct an evidence function that satisfies the standard desiderata for such functions. It is grounded in a discrepancy measure that compares competing models in light of the data and evaluates their relative distance from the unknown true data-generating process. Importantly, an evidence function needs not cross any threshold to count as “evidence”; rather, it provides a comparative measure of the distance between models and their distance from the truth.

An evidence function is public; its values are open, transparent, and available for all to inspect. At the same time, it is portable and shareable among investigators. Evidence is accumulable and can be combined to support inferences used to adjudicate between competing models. The evidence function should ideally be consistent, meaning that with sufficient data we will identify the true model

### 2.2. Prediction Goal

When the goal is prediction, the focus is on learning from past events to anticipate future occurrences with stability and reliability. In this context, CEMC evaluates the comparative evidential impact of data on competing models, aiming to estimate their predictive accuracy—how close each model comes to approximating the data-generating process.

To this end, AIC is employed. AIC is calculated as

−2*ln*(L) + 2*k*.

Here, AIC uses maximum log-likelihood estimate (*ln*(L)) and k is the number of adjustable parameters. AIC recommends selecting the model with the lowest AIC value. As demonstrated by Akaike, when estimating predictive accuracy, what matters is not the ground truth but the relative evidential proximity of models to the truth. This comparative framework suffices to evaluate how well models predict future data.

### 2.3. Explanation Goal

When the goal is explanation, the focus shifts from studying relationships between variables to uncovering causal relationships from observed data, emphasizing underlying systems and interactions. Here, CEMC applies the Schwarz Information Criterion (SIC) [15] or Bayesian Information Criterion (BIC) to evaluate models. The BIC is given by

−2*ln*(L) + *k*.

Here, BIC uses maximum log-likelihood estimate and *k* is the number of adjustable parameters. BIC penalizes model complexity more strongly than AIC, making it more conservative and less prone to overfitting. It is suited to explanation tasks where the goal is to identify simpler, more interpretable causal relationships/structures.

### 2.4. Epistemic Utility

CEMC treats model selection as an epistemic action, evaluated through epistemic utility. By adopting “as an epistemic action,” we mean the choice we will make about a model from a set of models is based solely on data-driven considerations. Drawing a parallel with economic utility, epistemic utility is defined as the expected benefit of choosing a particular model. It is assessed by maximizing estimated predictive accuracy and minimizing the risk of misleading evidence—cases where a model appears reliable but is actually flawed.

While economic utility focuses on maximizing consumer benefit, epistemic utility concerns the reliability and informativeness of models. As with economic theory, overfitting and underfitting are epistemic risks. Simplicity and interpretability of models are considered epistemic virtues. CEMC allows for incorporating the cost of decision-making into the utility calculation.

It is prudent to discuss the definition of a utility function here. We begin with a general non-Bayesian utility function for comparing models by evidence strength and distance to truth. Let

M = the set of models under consideration,

w = the true world (or true data-generating process), and

E = the total available evidence/data.

We want a utility function U:M×E×{w}→R that assigns a numerical epistemic value to a model M based on its distance from the truth and the strength of the evidence supporting it. No probabilities are required. To construct such a utility function, we need two primitives:

(i) Distance-to-truth function (non-Bayesian, model-theoretic):

d(M,w)∈[0,∞)

This represents a model–world discrepancy measure, or anything that captures how far the model is from reality.

(ii) Evidence-strength function (non-probabilistic) is defined as S(M,E)∈R.

Here, “strength of evidence” is non-probabilistic. It may be measured using likelihood-ratio measures, distance-to-data functions (e.g., least squares, without probabilistic interpretation), or unnormalized evidence measures. In summary, general non-Bayesian utility function for comparing models based on evidence strength and distance to truth is any function of the form

U(M;E,w)=f(S(M,E), d(M,w)),

where

(a) S(M,E) measures the strength of evidence for model M,
(b) d(M,w) measures the model’s distance from the truth, and
(c) f is any function increasing in evidence strength and decreasing in distance to truth. No Bayesian subjective credences or probabilistic structures appear anywhere in this formulation.

### 2.5. Objective Probability via Empirical Frequency

CEMC uses objective probability grounded in empirical frequency, rather than degrees of subjective belief. It replaces agent-centric degrees of belief with the observed frequency of events. This allows CEMC to quantify the probability of misleading evidence, enhancing its robustness in both predictive and explanatory contexts.

The empirical frequency of events feeds into the model’s assessment of how likely it is to mislead. Thus, CEMC can still reason under uncertainty without invoking subjective probability.

### 2.6. Statistical Tools

Statistical tools are central to CEMC. The choice of tools depends on whether the goal is prediction or explanation. For prediction, CEMC uses likelihood ratios and AIC to compare models based on estimated predictive accuracy. A model with a better fit to the data-generating process, as measured by the maximum likelihood estimate (MLE), is considered to have higher epistemic utility.

For explanation, CEMC uses BIC, which selects models based on posterior probability and penalizes unnecessary complexity. Compared to AIC, BIC is more conservative and less prone to overfitting, although it may underfit by excluding relevant variables.

CEMC emphasizes awareness of these trade-offs. Overfitting increases the risk of noise being mistaken for signal, while underfitting risks missing important explanatory variables. The balance between predictive accuracy and explanatory power hinges on the chosen criterion.

### 2.7. Goals, Varieties of Evidence Functions, and Their Performance Capabilities

In the CEMC, AIC is employed to address the goal of prediction, while BIC is employed to address the goal of explanation. However, there is a plethora of evidence functions. Although the formal structure of evidence functions is relatively new, several evidence functions have long proved their mettle. Likelihood ratios and log-likelihood ratios, for instance, are accepted evidence functions. Other evidence functions include order consistent information criteria, such as Schwarz’s Information Criterion [15], also known as BIC, the consistent AIC (see [16]), and the Information Criterion of Hannan and Quin [17], abbreviated as HQIC. These information criteria are all functions of the maximized log-likelihood under the model at hand plus a penalty term. As a result, the difference in the values of a given information criteria between two models is always a function of the likelihood ratio. Because likelihood ratio is an evidence function satisfying all the above desiderata of an evidence function, maximum likelihood parameter estimation is an evidence procedure. For two competing simple statistical models, the likelihood ratio is the most efficient evidence function [10].

A family of information criteria, such as AIC and AIC_C_ (Akaikean Information Criterion corrected for small sample sizes), is said to be asymptotically efficient when their efficiency (a measure of how well they approximate a true value) approaches a theoretical maximum as the sample size increases infinitely. For every large dataset, an asymptotically efficient measure becomes practically as good as the best possible estimator. They are efficient in terms of prediction as these evidence measures are good at making the best estimated prediction. Consider AIC and the model consistency desiderata. AIC is not consistent in the first sense of the term. The population parameter consistency states that even though sample size increases considerably, tending toward infinity, AIC will not be able to choose the true/correct model. However, AIC is consistent in the second sense. The latter is the goal of minimizing the prediction error rate of a model. AIC as an evidence measure serves this goal both reliably and accurately as it maximizes the goal for which it is primarily proposed consistently [18].

Likewise, a family of information criteria, such as BIC and HQIC, is compact, and their elements are well-supported. They underfit the data. Underfitting decreases as the sample size increases. They can find the true model if the sample sizes increase provided the true model is inside the model set. If the true model is not inside the model set, then as the sample size increases, it will select the model closest to the true model. In a small sample size, HQIC behaves like AIC. If sample size increases, it becomes consistent. This family of information criteria including BIC and HQIC can address the causal relationship between variables. Thus, they are appropriate for serving the goal of explanation. We will explain this feature of BIC in the next section.

Revisiting the desiderata for evidence function, we find that not all information criteria, strictly speaking, can be construed as evidence functions properly as some evidence functions satisfy most of the desiderata but not all of them. There is a class of information criteria which suffers from this shortcoming of not obeying all of the features of an evidence function properly. They can be designated as minimum total discrepancy (MTD) forms [19]. “Minimum total discrepancy” is a notion applied to making an appraisal to the extent to which model or data aligns with a target set or trying for the closest possible agreement by minimizing the overall differences. In statistical modeling, the discrepancy between predicted values and actual values is usually minimized by using measures such as minimizing the mean squared error. This amounts to saying the model parameter that yields the smallest overall difference between prediction and observed data. MTD obeys desiderata (a to g), but not h. The AIC and the bias-corrected AIC (AICS [20], see ref. [18] for these discussions) are MTD criteria. These forms are not strictly evidence functions. This does not suggest they are mistaken criteria and should not be used evidentially. Even we apply some versions of them towards the prediction goal of the ECMC. The goal of these criteria is to select models to minimize prediction error, while the goal of some evidence functions is to both explore and understand underlying causal structure [16,19,21]. The consequence of this is that asymptotically, all MTD forms will overfit the data by tending to include variables with no real causal interactions with the response variables. However, in smaller sample sizes, the differences between them are not clear-cut. In model selection, the area under the (ROC) curve, abbreviated as AOC, tends to overfit in all sample sizes, while the AICc can have a stronger complexity penalty than the order that consists of forms such as BIC family.

## 3. Two Goals and Statistical Model Selection Criteria

We will discuss mostly two types of model section criteria, the Bayesian models and the Akaikean one to demonstrate how they function when asked to serve two types of goal, the explanation and prediction goals. We begin with the Bayes Theorem Criterion (BTC) which we advocated as a model selection criterion as a kindred spirit with the well-known BIC. Then, we will bring in the BIC followed by AIC. The primary goal is to show how model selection criteria, whether they are Bayesian or Akaikean, serve a specific goal in the Epistemic Utility Theory based on a comparison of models (or hypotheses). This section draws heavily from our previously unnoticed work [22].

### 3.1. Bayes’ Theorem Criteria (BTC)

One way to become clearer about how our three criteria select models or hypotheses is to focus on a particular example. This example has to do with the prediction of gas consumption at different temperatures based on past data concerning the correlation between consumption and temperature. Those who live in colder climates must face this problem at one time or another, especially if they heat their home with natural gas.

Sue is one such woman. The amount of gas required to heat her home depends on the outdoor temperature; the colder the weather, the more gas is consumed. If the family’s habits, the insulation of the house, and other relevant factors remain unchanged, Sue should be able to predict gas consumption based on the outdoor temperature. Reflecting on this example, we can analyze it in terms of causal explanation or prediction of future data.

From a causal perspective, we assume that all other relevant factors—such as the family’s habits and the house’s insulation—remain constant. This is equivalent to holding those variables fixed, effectively setting them to zero in the analysis. Under these assumptions, the outdoor temperature becomes the sole relevant causal factor for the household’s monthly gas consumption. This allows us to explain how temperature causally affects gas usage.

In contrast, when the goal is prediction, the same assumptions about constancy of other factors still apply. However, the aim is not to explain why gas consumption changes, but simply to forecast how much gas will be used based on the outdoor temperature. Thus, although the same variables are held fixed, the conceptual goal differs. The causal explanation seeks to identify the effect of temperature, whereas prediction focuses on accurately estimating consumption without attributing causality.

The usual need for heating is measured in degree days. One degree day is accumulated for each degree the average daily temperature falls below 65 degrees Fahrenheit. An average temperature of 20 degrees F., for example, corresponds to 45 degree days. Table 1 presents historical data for one season. In Table 1, the explanatory variable, x, is heating degree days for the month, and the response variable, Y, is gas consumption per day in units of hundred cubic feet.

Sue’s goal is to use these data to predict gas consumption at different temperatures. To do this, she needs to find a relationship between x and Y. The most common technique for fitting a line to data is known as the method of least squares. Consider Table 1.

The curve fitted to the data in Table 1 is commonly referred to as the regression line. We posit that each $Y_{i}$ is linked to its corresponding $x_{i}$ through the linear relationship:

$$\;\;Y_{i}=\alpha_{0}+\sum_{j=1}^{k}\alpha_{j}x_{i}^{j}+\epsilon_{i\;},\mathrm{for}\;i=1,2,\ldots ,n,$$

where n denotes the sample size; $\alpha_{j}$; *j* = 1, …, *k* are the unknown regression coefficients; *k* specifies the order of the polynomial model; and the errors $\epsilon_{i\;}$, *i* = 1, …. *n* are assumed to be independent Gaussian random variables with mean zero and variance σ^2^.

Each possible regression model gives rise to a hypothesis—$H_{1}$, $H_{2}$, or $H_{3}$—and these hypotheses are mutually exclusive by construction. They arise by specifying the polynomial order as 1, 2, or 3, yielding

$$H_{1}:E\left(Y|x\right)=\alpha_{0}+\alpha_{1}x;\;H_{2}:E\left(Y|x\right)=\alpha_{0}+\alpha_{1}x+\alpha_{2}x^{2};\;\;H_{3}:E\left(Y|x\right)=\alpha_{0}+\alpha_{1}x+\alpha_{2}x^{2}+\alpha_{3}x^{3}.$$

Here, E(Y∣x) denotes the conditional mean of Y given x. Declaring these hypotheses mutually exclusive simply means that in $H_{k}$, the coefficient of $x^{k}$ is presumed to be non-zero. Under $H_{1}$, the least-squares prediction is

$$\hat{Y}=1.22+0.20\times 15.3=4.33$$

hundred cubic feet. For $H_{2}$, the forecast becomes

$$\hat{Y}=1.09+0.22\times 15.3-0.0005\times 15.3^{2}=4.39$$

hundred cubic feet per day—already closer to the historical value of 4.5. Employing the cubic model $H_{3}$ produces $\hat{Y}=4.45$, which comes even nearer to the historical benchmark. As is often the case, raising the order of a polynomial regression improves its fit to the existing data.

A familiar metric for judging such fit is the likelihood function, evaluated at the estimated parameters. Let the maximized likelihood under hypothesis $H_{k}\;$be

(1) $${\hat{L}}_{k}={max}_{\sigma^{2};\alpha_{0},\ldots ,\alpha_{k}}L_{k}(\sigma^{2};\;\alpha_{0},\ldots ,\alpha_{k}|H_{k};Y_{1},\;\ldots ,Y_{n}),$$

where $L_{k}$ denotes the likelihood itself. Since larger polynomial orders can mimic the data more closely, ${\hat{L}}_{k}$ increases with k. This might tempt one to conclude that higher-order models automatically yield superior predictions. Yet the reality is more subtle: overly flexible models tend to overfit and often give worse forecasts than more parsimonious alternatives.

Thus, the challenge of selecting an appropriate model resurfaces in the Sue example. To address it, we introduce the Bayes Theorem Criterion (BTC), an approach grounded in Bayes’ theorem. As shown in [23], if one adopts certain non-informative priors on $\sigma^{2}$ and on $\alpha_{j}$ for j=0,…,k, then the posterior probability $Prob\left(H_{k}|\mathrm{data}\right)$ becomes proportional to its prior $Prob\left(H_{k}\right)\;$multiplied by the maximum likelihood value ${\hat{L}}_{k}$.

In our proposal, prior probability measures the simplicity of a hypothesis. A hypothesis achieves a higher probability than its contenders, ceteris paribus, if it has fewer parameters. In contrast, we say that the likelihood function measures the goodness of fit. A hypothesis with more parameters generally has a higher likelihood than the one with fewer parameters. This is because of the way we characterize the log-likelihood in (1). If we assume (1), then as the order of polynomial model increases, the maximized likelihood increases. Given the prior probability and likelihood function of a hypothesis, we obtain its posterior probability. BTC recommends choosing the hypothesis that has the highest posterior probability as making the best trade-off between simplicity and goodness of fit.

The notion of prior probability plays a crucial role in BTC. In its application, two factors, formal and non-formal, determine the prior probability of the hypotheses, Prob($H_{k}$). The formal factor is paucity of parameters and this factor orders hypotheses with respect to simplicity. Recalling the three hypotheses, $H_{1}$, $H_{2}$, and $H_{3}$, where the order of the polynomial model is denoted by the subscript. A hypothesis achieves a higher probability than a competitor, ceteris paribus, if it has fewer parameters. That is, Prob($H_{1}$) > Prob($H_{2}$) > Prob($H_{3}$).

Restricting the prior probabilities of the hypotheses to satisfy Prob($H_{i}$) > Prob($H_{j}$) whenever *i* < *j* is not sufficient to determine the values of these probabilities. Non-formal factors, especially epistemological and pragmatic factors, play a key role in arriving at the specific values of prior probabilities. Given what we have said so far, we consider $H_{1}$ as the simplest hypothesis and assign the highest prior probability, 1/2, to it, because of epistemological/pragmatic considerations. $H_{2}$ is assigned 1/4 and $H_{3}$ 1/8. Recall that we considered only three hypotheses. The rest of them are lumped together. This is called the catch-all hypothesis, denoted by $H_{C}$, and is assigned 1/8. In short, BTC says, choose the one that maximizes posterior probability, that is, maximize

(2) $$Prob(H_{k}|data)\;\propto \;{\hat{L}}_{k}\times Prob\left(H_{k}\right).$$

In calculating epistemic utility within the CEMC framework, we must consider both the evidential support for each hypothesis and the utility associated with acting on that hypothesis. Specifically, we compute the epistemic utility for each hypothesis by multiplying its evidential support with its corresponding utility. We then select the hypothesis with the highest epistemic utility among the competing options.

The evidential support is typically represented by the log-likelihood of the hypothesis, while the utility component reflects the complexity of the hypothesis—measured, for example, by the number of adjustable parameters. A more complex hypothesis tends to have a lower prior probability, thereby reducing its overall epistemic utility. This consideration underlies the interpretation of Equation (2) as a shorthand for calculating the epistemic utility of each hypothesis.

### 3.2. Akaikean Information Criterion (AIC)

In the philosophy of science, Malcom Forster and Elliott Sober pioneered the application of AIC to scientific inference, which will follow in this section [24]. In the curve-fitting problem, according to Forster and Sober, we would like to choose the curve that is closest to the true curve. Their goal is to measure the closeness of a family of curves to the truth. They use AIC to achieve this goal. The theory underlying AIC assumes that there is a true distribution of the observable random variables. Call this distribution *f*(**Z**|θ) (or *f*) where **Z** is a vector of observable random variables (i.e., future data) and θ is a vector of unknown parameters (e.g., $\sigma^{2};\;\alpha_{0},\alpha_{1},\ldots .,\alpha_{k}).$ Under $H_{k},\;$an approximation to the unknown distribution, f, is desired. In the Akaike approach, one approximates f in two steps. First, a sample of data, say **Y**, is observed and $\theta_{k}$, the maximizer of $f_{k}(Y\;|\theta )$ with respect to θ, is computed. Second, we approximate the unknown density, f, by ${\hat{f}}_{k}=\;{\hat{f}}_{k}\;(Z|\theta_{k}).$

Forster and Sober [24] explain how an agent can predict new data from old data with the help of these steps. First, an agent uses the available data to obtain the *best-fitting* members of the family. Then, this best-fitting member of the family is used to provide what new data will look like. The question is now how well the curve in the family that best fits the data will do in fitting the new data.

In most cases, *f*, the true distribution, is unknown. When *f* is unknown, we use ${\hat{f}}_{k}\;$in which the value of *k* is chosen to minimize the distance between *f* and ${\hat{f}}_{k}$. Akaike showed in [14] that the measure of distance between *f* and  ${\hat{f}}_{k}$ is

$$D\left(f,{\hat{f}}_{k}\right)=\frac{1}{n}\;\left[\ln\left(\frac{f}{{\hat{f}}_{k}}\right)\;f\;dz\right].$$

Choosing the family of curves which minimizes the distance between f and ${\hat{f}}_{k}\;$is equivalent to choosing the family of curves which maximizes the quantity

(3) $$\;A\left(f,{\hat{f}}_{k}\right)=\frac{1}{n}\;E\left[\ln\left({\hat{f}}_{k}\right)\;f\;dz\right],$$

where the expectation is taken with respect to the distribution of the estimator ${\hat{\theta }}_{k}$. Ref. [24] call $A\left(f,{\hat{f}}_{k}\right)$ the predictive accuracy of the kth family of curves. Akaike showed that under certain conditions, maximizing the predictive accuracy of a hypothesis is equivalent to choosing the hypothesis that maximizes

(4) $$\mathrm{AIC}=\ln\left({\hat{L}}_{k}\right)-k,$$

where k represents the number of parameters. Like BTC, AIC involves two key components: the log-likelihood and the penalty function. The penalty function depends on the number of parameters in each model. Unlike BTC, AIC is completely objective and does not rely on an agent’s subjective degree of belief. To compute the epistemic utility of each hypothesis using AIC, the log-likelihood captures the evidential strength of the hypothesis, while the penalty function accounts for its complexity. Complexity is measured by the number of parameters in the hypothesis. To calculate the epistemic utility, we subtract the penalty (number of parameters) from the log-likelihood for each hypothesis. The hypothesis with the largest AIC value among the competitors is then selected.

### 3.3. Bayesian Information Criterion (BIC)

Historically, AIC preceded BIC. To understand BIC, it is better to compare it with AIC. Recall that AIC suggests maximizing the likelihood function for each alternative model. After taking a logarithm of the likelihood function, it subtracts the number of parameters in the corresponding model. At the end, AIC chooses the model that has the largest numerical value. The criterion proposed by Schwarz (BIC) chooses the model for which

(5) $$\mathrm{BIC}=\ln\left({\hat{L}}_{k}\right)-\frac{1}{2}k\ln\left(n\right),$$

is the largest. Thus, BIC differs from AIC in that the dimension of the model is multiplied by *ln*(n). For the number of observations usually found with economic data, BIC favors a lower-dimensional model than AIC.

Both BTC and BIC are Bayesian in nature. That is, both begin with priors over the parameters. However, there is a difference between them insofar as the priors are concerned. To obtain the posterior probability as in (2), Ref. [23] adopted a Gaussian prior on the vector of regression coefficients. The prior variance of the regression coefficient was assumed to be of order of magnitude $\frac{1}{n}$. If the prior on the regression is redefined so that it does not depend on *n*, then the posterior probability of *H_k_*, given the data, can be shown to be

(6) $$Prob(H_{k}|data)\;\propto \;{\hat{L}}_{k}\times Prob\left(H_{k}\right)\times n^{-k/2}.$$

The right-hand side of (6) is equivalent to Schwarz’s BIC [15], provided that Prob(*H*_k_|data) is not a function of n. There is no difference between (5) and (6), except that (5) expresses BIC in log scale. There is another difference between BIC and BTC. Although BIC and BTC recommend the choice of that hypothesis which maximizes the posterior probability, BIC applies only to large-scale samples, whereas BTC is concerned with both small and intermediate sample sizes. However, as the sample size increases, BIC and BTC tend to converge in the limit. In BIC, the epistemic utility is calculated the same way as in BTC, where it is evaluated for each hypothesis individually. Hence, its philosophical theme will be the same as the BTC.

### 3.4. A Comparison Between AIC and BTC and Two Goals of CEMC

Recall that the Bayesian criteria BTC and BIC choose the hypothesis that maximizes posterior probability, whereas AIC chooses the hypothesis that maximizes “predictive accuracy.” The three approaches are summarized in Table 2 using the Sue data. Consider BTC and AIC. BTC says that Prob($H_{i}$|Y) is proportional to $\theta_{i}\times \;L\;$, where $\theta_{i}\;$represents priors and L represents the likelihood function. Alternatively, one can say that one can choose the model for which log(Prob($H_{i}$|Y)) = ln $\theta_{i}$ + In L + c is large. Here, c is a constant.

Consider AIC that says maximize *lnL*−k, where *lnL* is the log-likelihood of the sample and *k* represents the number of parameters. The purpose of the penalty function, -k, is to penalize the value of the function when new parameters are added.

Again, we observed that the purpose of AIC is to estimate the predictive accuracy of competing models. In contrast, the purpose of various Bayesian criteria (such as BTC and BIC) is to explain either causal relationships between variables or the causal mechanisms underlying the data-generating process. Both information criteria—AIC and the Bayesian Information Criterion—are composed of a maximized log-likelihood function minus a penalty term. The log-likelihood reflects the strength of the data’s support for the competing models, while the penalty function accounts for the bias introduced by adding new parameters; importantly, the penalty function represents different utilities in each criterion.

To derive model-based epistemic utility functions for each model, we must incorporate both the evidential strength of the data, and the comparative utilities associated with each model. In the “Sue” example, we implicitly seek to identify the causal relationship between heating degree days and monthly gas consumption using the Bayesian Information Criterion. In contrast, the application of AIC in the same example aims to estimate average gas consumption for coming months based on past data.

This difference in goals could explain why Table 2 yields different values for AIC and the Bayesian Information Criterion. However, it is noteworthy that both criteria—whether Bayesian or AIC-based—select model $H_{1}$ as the best. In the Sue example, we discuss how different model criteria function without demonstrating that each might select different best models among the contenders.

For a given data set, the predictive goal may favor one model, while the explanatory goal may favor another. Accordingly, the decision procedure is straightforward: use AIC for prediction and use BIC/BTC for explanation. More succinctly, if one insists on a single decision procedure, then one should apply BTC/BIC, since doing so can secure both explanatory and predictive aims.

In the next section, we will highlight the motivation behind Kepler’s search for a planetary model, particularly his first planetary law, to show that his approach aligns more closely with the BIC framework than with AIC.

### 3.5. Kepler’s Search for Planetary Laws and Bayesian Information Criteria

A milestone example from the history of physics illustrates and, in the process, should help clarify the distinction between predictive and explanatory goals embedded in the CEMC framework. It has to do with Kepler’s search for what is now known as the first law of planetary motions. The search was complex, marked by several fits and starts, and spanned nine years. But a brief sketch of it should suffice to make our point.

On the standard account, Kepler wanted to provide a curve-fitting analysis of the planetary orbits, trading off simplicity and goodness-of-fit desiderata in the usual way. His data set consisted of the naked-eye observations of planetary motions, in particular that of Mars, made by Tycho Brahe over a long period of time. Although he tried out a number of different hypotheses along the way, Kepler eventually reduced them to three: the path of a planet is circular, elliptical, and ovoid. His equations for these figures are, respectively:

1. Circle: $x^{2}/r^{2}+y^{2}/r^{2}=1.$
2. Ellipse: $x^{2}/a^{2}+y^{2}/b^{2}=1.$
3. Ovoid: $x^{2}/a^{2}+y^{2}/b^{2}+z^{2}/c^{2}=1.$

As the equations of these three figures indicate, the circular equation is simplest (one 4adjustable parameter, *r*), the elliptical more complex (two adjustable parameters *a* and *b*), and the ovoid most complex (three adjustable parameters *a, b,* and *c*). Convinced initially that the circular (Copernican) hypothesis must be correct (in part on theological grounds; God would have chosen the simplest possible orbits for the planets), he spent a great deal of time trying to fit it to the data. But this effort failed. Given that the other two hypotheses are (within an order of approximation) equally likely, he chose the simpler, which in this case is the elliptical.

On his curve-fitting account, Kepler’s model is kinematic and his goal predictive. It thus lends itself to the Akaike Information Criterion on which the model selected has the lowest AIC value. As we have already noted, what matters is not truth per se, but the relative evidential proximity of models to an idealized surrogate for it.

But there is more to Kepler’s story [25]. Which is to say that he did not rest content with predictive accuracy, however precise it might be. For it was a presupposition of his world view that an acceptable hypothesis must also be true simpliciter. This in turn required that the hypothesis be explanatory as well, i.e., must also identify the causal factors behind and not merely the correlations between the observational data by way of their emphasis on prior probabilities captured in Bayesian information criteria. In the process of reconstructing the Kepler case, we assume the way he approaches the problem is to begin with circles as the simplest and gradually make geometrical shapes more complex. That is, from a circle, to an ellipse, to ovoid shapes. His initial belief, according to the Bayesian account, was that a circle, given the data and his background information, was most likely to be the best causal explanation for how planets could move around the sun, with the next geometrical shape being gradually less likely, and so on. This is how he might have inferred on conceptual ground which shape is the most likely causal explanation for planets pivoting around the sun. Though he is a theoretician, he is both an astronomer and physicist. Consequently, he cannot afford to overlook data gathered from Tycho’s observations. Consider some basics of Bayesianism regarding Kepler’s search for the first planetary law.

First, the distribution of prior probabilities over the hypotheses to be compared must sum to 1. That is, application of Bayesian information criteria to a comparison among two or more hypotheses presupposes that one of them is true, i.e., the true model must be in the model set.

Second, an agent’s choice of hypotheses is prior to their comparison and can include a restriction to causal hypotheses. If causal, then by the same token they will be explanatory.

These two considerations come together in a closer look at the way in which Kepler proceeded. On the one hand, he implicitly assumed that either the elliptical or ovoid hypothesis was true since they were the only hypotheses consistent with the data. On the other hand, he also implicitly assumed that physical in contrast to merely observational considerations would serve to determine which hypothesis was in fact true. Kepler was able to calculate that a central force emanating from the sun (as a Copernican he thought that the sun was the locus of central forces acting on the planets) would have to obey an inverse square law and that only an elliptical orbit was consistent with such a law. Kepler’s underlying assumption is that only a dynamic account of an orbit in terms of the force capable of explaining its shape could establish that its elliptical shape was in fact the true one. In a nutshell, truth was the aim and an explanation in terms of causal factors could reach it.

In a generally Bayesian way, he was the first to chart this course. His aptly named *Astronomia Nova* has its subtitle “Based on Causes, or Celestial Physics, Brought Out by a Commentary on the Motion of the Planet Mars.” Kepler wrote, “the novelty of any discoveries and the unexpected transfer of the hole of astronomy from fictitious circle to nature causes were most profound to investigate, difficult to explain, and difficult to calculate, since mine was the first attempt.” It was perhaps more in this way, by insisting that the true account of orbital reality required a physical dynamic in addition to a traditional geometrical kinematics, that he forged a new path forward for astronomy.

Thus, although it was a historic breakthrough in the history of astronomy to do so, Kepler’s case parallels the Sue example discussed earlier as concerns. He began by asking how the motion of the planets could be predicted. In this, he was at one with his predecessors in the history of astronomy, whose sole interest was in answering a “what?” question: given data about the position of a planet at any one time, what will be its position at any future time? Sue’s similar sort of goal was to predict her home’s gas consumption as a function of the outdoor temperature based on past data correlating the two. In Kepler’s case, positing elliptical and ovoid orbits because of past observational correlations sufficed to make adequately precise predictions. But in both cases, the “what?” question was followed by a “why?” question: why does gas consumption vary as the temperature changes? Why do the planets trace particular orbital paths? In both cases, the goal was a causal explanation. His answer was that only an elliptical orbit was consistent with an explanatory inverse square force, just as in our schematic example it was demonstrated that temperature variation and not such other variables as family habits and house insulation was responsible. His causal answer sufficed to demonstrate that the first but not the second was true. First correlation, then causality, which is to say that Kepler’s way forward went beyond the AIC to something like the BIC framework.

## 4. Revisiting Model Selection Criteria and Their Two Goals Served in One Criterion

Among various possible goals of comparative model-based evidential decision accounts, we focused only on two goals: (i) to predict and (ii) to explain either the causal relationships between variables or the causal mechanism of the phenomena at stake. We will show here that Bayesian information criteria can serve both goals the best.

At least one major difference in the formalisms of AIC and BTC types of model selection criteria is that the former applied a two-step process to estimating future data. The classical approach first uses data to estimate the maximum likelihood (MLE) and then uses the MLE to predict future observations. In contrast, BTC-type criteria employ the predictive density to estimate how future data are likely to behave. Suppose the objective is to maximize predictive accuracy. While this goal is reasonable, one may question why ${\hat{f}}_{k}(Z|{\hat{\theta }}_{k})$ is chosen as the approximating density. Reference [26] demonstrated that if a prior distribution is assumed for the unknown parameter vector θ, the density that maximizes predictive accuracy is $f_{k}(Z|Y)$, rather than ${\hat{f}}_{k}(Z|{\hat{\theta }}_{k})$. In other words, the optimal predictive density for future data Z is the conditional density of Z given the observed data Y. This is known as the posterior predictive density.

Moreover, the posterior predictive density is only minimally affected by the choice of prior for θ. For moderate to large samples, the specific prior has little influence. For example, when selecting a family of regression models, the posterior predictive density, to order $1/\sqrt{n}$, is multivariate t. In general, the gain in predictive accuracy (Equation (4)) from using $f_{k}\left(Z|Y\right)$ instead of ${\hat{f}}_{k}\left(Z|{\hat{\theta }}_{k}\right)\;$is approximately $\frac{k}{2}\;[1-\ln\left(2\right)]$ ≈ 0.15 *k*. This result assumes that the dimensions of the future data Z and the observed data Y are equal. The Akaikean Criterion, when modified by substituting $f_{k}\left(Z|Y\right)$ for ${\hat{f}}_{k}(Z|{\hat{\theta }}_{k})$, selects the hypothesis that maximizes ln (${\hat{L}}_{k}$) − 0.85 *k*.

If one assumes that the true model is contained within one of the approximating families under consideration, an even more effective approach is possible. In this setting, Ref. [27] proposed a Bayesian prediction criterion for selecting models based on expected predictive accuracy. Ref. [28] applied this method to regression models, and his simulation results indicated that substantial improvements in predictive accuracy over AIC are achievable. Apparently, Ref. [29] does not oppose this approach but is reluctant to specify a prior for the unknown parameters—a limitation that appears unnecessary. Roschenhofer, for instance, adopted a non-informative prior and still achieved improvements over the AIC.

Let us emphasize one important finding of Section 2 concerning the issue of predictive accuracy of family curves and which approach, BTC (i.e., our Bayesian approach) or AIC, is preferable. We showed that if maximizing estimated predictive accuracy is the goal of some accounts, then the density that maximizes predictive accuracy is $f_{k}(Z\;|\;Y\;)\;$rather than ${\hat{f}}_{k}(Z\;|\;{\hat{\theta }}_{k})$, which is central to Akaikean Information Criterion. This density is nothing but the posterior predictive density core of any Bayesian account including ours [24,30].

One possible concern needs to be addressed. The superior performance of the Bayesian Information Criterion in terms of explanation and prediction is often attributed to differences in how AIC and Bayesian inference operate. As discussed previously, the AIC framework follows a two-step process: first, the maximum likelihood estimate (MLE) is computed from the data; second, this MLE is used to estimate future data. It is sometimes claimed that Bayesian inference outperforms AIC in prediction because it avoids this two-step process, thereby reducing the chances of error.

However, this rationale for the superiority of Bayesian methods is misplaced. If the priors chosen in Bayesian inference are significantly different from the true priors, then the resulting inferences can be highly unreliable—although the Bayesian approach uses a single-step calculation. In such cases, Bayesian calculations may perform no better than or worse than those based on AIC.

It is important to emphasize that when it comes to maximizing estimated predictive accuracy, the choice of priors plays little to no role. This is a robust result, underscoring the strength of the Bayesian Information Criterion in both explaining the influence of one variable on another and estimating the predictive accuracy of a hypothesis.

## 5. Evidential Interval-Based Decision-Theoretic Accounts

We will briefly discuss two such decision-theoretical accounts: (i) Kyburg’s evidential account and (ii) our Weak-Dominance-based decision principle (WDP) which draws its inspiration from Kyburg’s account. There are at least three similarities between the two accounts we will broach here. (a) Both are interval-based evidential probability. (b) Neither the goal to explain nor to predict has more than a trivial meaning here, as we will address. And (c) in both cases, an agent’s rational belief about an event should be guided by its interval-based probability when there is no single probability of the event being available. One significant difference between both of these accounts and the previous CEMC is that unlike the latter, where the account makes the place of an agent’s belief redundant, these two accounts emphasize the theme that an agent’s rational belief must be proportional to its interval-based probability, hence they make it important for one’s belief to conform to how much evidence is associated with it. Otherwise, one’s rational degree of belief needs to be diminished or relinquished.

### 5.1. Kyburg’s Evidential Decision Theory

Kyburg’s evidential decision theory is a philosophical and formal approach to decision-making under uncertainty. It incorporates statistical evidence of an event in forming an agent’s belief in it, rejecting single-valued probability theory. Probability of an event is measured in terms of its intervals, for example, [0.4, 0.9], rather than by precise probability value. These intervals reflect bounds on an agent’s *rational belief* derived from observed frequencies in appropriate reference classes. When one wants to make an inference based on probabilities associated with those events, these probabilities are determined by the most specific reference class with known statistical data. E.g., if 90% of $E_{1}\;$events are P, 40% of broader class $E_{2}$ are P, and $E_{1}\subseteq E_{2}$, the probability of a specific $E_{1}$ event being P is [0.4, 0.9]. It avoids subjective prior probabilities, relying solely on empirical frequences. This distinguishes his account from the Bayesian account that incorporates prior probabilities defined over events (see [31] for an attempt to reconcile his evidential theory of probability with objective Bayesianism). According to Kyburg, probability represents a necessary relation between a set of accepted sentences and a given sentence. For him, this relation does not assign a single real number to represent the probability that a sentence S is true relative to a knowledge corpus K. Instead, it characterizes the probability of S relative to K as the interval [p,q] if and only if:

(i) S is known in K to be equivalent to a sentence of the form “a is an element of set b.”
(ii) “a is an element of c” is a sentence in K.
(iii) The proportion of cs that are bs is known in K in the interval [p, q].
(iv) Relative to K, a is a random member of c with respect to b.

Kyburg’s central decision rule, Principle III, states the following:

*The decision maker should reject any choice a_i_ for which there exists an act a_j_ whose minimum expected utility exceeds the maximum expected utility of a_i_.*

He further proposes the Maximin principle, which recommends maximizing one’s minimum gain. Combining Principle III with Maximin often yields sound decisions.

### 5.2. The Weak Dominance Evidential Decision Principle

We propose a decision principle within Kyburg’s probabilistic framework [8]. Like his theory, our approach uses probability intervals. The probability of a proposition S being true relative to a knowledge corpus K belongs to [p,q]. Complete knowledge reduces the interval to a point; incomplete information widens it. Complete ignorance is represented by [0,1] [9].

To connect degrees of belief with actions, let U be a real-valued utility function over sentences. As probabilities are intervals, expected utilities are also intervals. If S has probability [p,q] and utility U, its expected utility is [Up,Uq].

Maximizing intervals as if they were points is not meaningful. Kyburg suggests a rational alternative: one should not choose an action whose maximum expected utility is less than the minimum expected utility of some other action. This eliminates many irrational acts. We extend his principle with additional conditions, forming the **Weak Dominance Principle (WDP):**

*J weakly dominates I if choosing J always yields at least as good an outcome as I, and there exists at least one scenario where J yields a strictly better outcome than I.*

We illustrate WDP using a utility matrix with two probability distributions $P_{1}$ and $P_{2}$. Let the matrix be **M**:

States Probability Distributions

$$S1\;S2\;P_{1}(S1)=0.4$$

$$\mathrm{Acts}\;P_{1}(S2)=0.6$$

$$A1\;1-1\;P_{2}\left(S1\right)=P_{2}(S2)=0.5$$

A2 −1 1

A3 0 0

Expected Utility (A1) = [−0.2, 0]

Expected Utility (A2) = [0, 0.2]

Expected Utility (A3) = [0, 0]

According to Kyburg, any choice is rational, as his principle does not eliminate any options. WDP, in contrast, selects A2 as rational because it is never worse than alternatives and strictly better in some scenarios. Thus, A2 is the natural choice.

#### Ordering Relationship of the Decision Rule

Under WDP, for closed intervals I and J, if min (I)≤min (J), max (I)≤max (J), and either min (I)<min (J) or max (I)<max (J), then the act corresponding to J should be chosen. This induces a strict partial ordering: irreflexive and transitive on the intervals (see [31]).

WDP induces a lattice structure: for any two elements a and b, there exists a least upper bound (lub) and greatest lower bound (glb).

Strict preference and indifference are determined as follows:

1. If two intervals are disjoint and max (I)<min (J), choose the act corresponding to J.
2. If intervals are not disjoint and have the same maximum, choose the act with the higher minimum.
3. If intervals are not disjoint and have the same minimum, choose the act with the higher maximum.

An agent is indifferent if max (I)=max (J) and min (I)=min (J). Many acts are incomparable, meaning neither preference nor indifference applies.

All decision principles should satisfy an **adequacy criterion**. The **mixture condition** states that if an agent is indifferent between two acts, they should also be indifferent to a third “mixed” act that selects one act on heads and the other on tails of a fair coin.

For example, if $A_{1}$ and $A_{2}$ have utilities [1,0], a mixed act $A_{3}$ yields:

S1 S2

A1 1 1

A2 1 0

A3 1/2 ½

Here, $A_{3}$’s utilities are expected values of a fair coin paying 0 or 1. After applying the mixture condition, the agent is no longer indifferent among the three acts. While $A_{1}$ and $A_{2}\;$remain pairwise indifferent, neither is indifferent to $A_{3}$. $A_{3}\;$has the highest minimum (1/2), whereas $A_{1}$ and $A_{2}$ have higher maximums. No single action is uniquely rational.

Thus, $A_{1}$, $A_{2}$, and $A_{3}$ are **incomparable**, leaving the choice to the agent’s preference. Many acts, particularly those with the same minimum or maximum but differing in the other bound, fall into this category. For instance, $A_{1}=[1,100,00]$ and $A_{2}=[100,120]$ provide no unique solution, nor is it rejected by principle. This realism reflects the principle’s ability to capture the true state of nature.

One fundamental respect in which the Weak Dominance Principle (WDP) differs from Kyburg’s decision theory is that when an agent has a single probability distribution over an event, WDP reduces to a Savage-style decision theory. In contrast, Kyburg’s theory does not exhibit this convergence.

Aside from a trivial sense, neither Kyburg’s nor other interval-based evidential accounts are clearly oriented toward the goals of explanation or prediction. In a particular decision situation, **M**, the WDP, yields a more intuitive and arguably superior decision than Kyburg’s theory. One might even argue that WDP better anticipates the decisions it will or will not recommend in other possible scenarios. Nevertheless, neither WDP nor Kyburg’s theory comes close to fulfilling the explanatory or predictive aims that CEMC is designed to address. Thus, apart from a superficial resemblance, these interval-based theories fall short of CEMC in terms of explanatory and predictive power.

We conclude this section by revisiting the desiderata for an evidence function and evaluating the extent to which these interval-based decision-theoretical accounts satisfy them. Mapping one framework onto the other is difficult, though they share a kindred spirit. These accounts meet conditions (a) through (c) in Section 2.1: Desiderata for evidence functions, but they do not explicitly address how to estimate the relative distance between models and the data-generating mechanism. It is also unclear whether evidence is public in these frameworks, as the evidential value of an event appears to depend on its reference class and the epistemic agent possessing the relevant information.

Furthermore, neither theory satisfies the desideratum that evidence should be **portable**, since this notion remains ambiguous in the context of these theories. However, both theories satisfy condition (f): when two datasets pertain to the same pair of models, the evidence should be combinable with respect to the models or hypotheses in question. They also appear to satisfy condition (g): evidence should not depend on the personal idiosyncrasies of model-specific information.

Finally, the two notions of **consistency** that we defined earlier have little bearing on what these interval-based evidential accounts are intended to address. While we have emphasized the importance of explanation and prediction, and while each model selection criterion discussed in Section 2 is intended to serve one of these goals, no such obligation is borne by these interval-based decision theories.

## 6. Conclusions

The paper revolves around three central ideas. First, we outlined a Comparative Evidence-Based Epistemic Utility (CEMC) framework for a non-Bayesian epistemic utility theory, although we drew on certain ideas from Bayesian versions of information criteria. Among the various possible goals of this framework, we focused on two: (i) explanation and (ii) prediction, to illustrate how the CEMC operates. Second, we introduced an interval-based evidential decision theory, accompanied by what we called the Weak Dominance Principle (WDP), drawing insights from interval-based approaches. Third, we compared the CEMC with both the interval-based decision theory and WDP.

We discussed how Bayesian versions of information criteria are typically suited to the goal of explanation, whereas the Akaike Information Criterion (AIC) is more aligned with prediction. Interestingly, in our empirical data analysis, both the Bayesian and Akaikean evidence functions selected the same hypothesis as best—a result not uncommon for particular phenomena. Furthermore, we showed that the Keplerian search for planetary laws can be both reconstructed along the Bayesian line and explained using Bayesian information criteria, due to the causal forces between the sun and planets—something AIC cannot capture. In this sense, Bayesian information criteria can serve both explanatory and predictive goals satisfactorily.

Finally, we examined Kyburg’s interval-based decision theory and WDP as methods for selecting the best option in a decision-making context. In comparing CEMC with Kyburg’s approach and WDP, we found that, unlike CEMC, these latter frameworks are not designed to explain or predict phenomena in a substantive way.

## Figures and Tables

**Table 1:** Gas consumption and temperatures over different months where x represents heating degree days for one season and Y represents the gas consumption per day in hundreds of cubic feet.

Variable	Month								
	Oct.	Nov.	Dec.	Jan.	Feb.	Mar.	Apr.	May	June
**x**	**15.6**	**26.8**	**37.4**	**36.4**	**35.5**	**18.6**	**15.3**	**7.9**	**0.0**
**Y**	**5.2**	**6.1**	**8.7**	**8.5**	**8.8**	**4.9**	**4.5**	**2.5**	**1.1**

**Table 2:** Comparison of BTC, BIC, and AIC for the Sue data.

Approach	General Criterion	Prior	Likelihood (Log Scale)	Applied to Sue Data		
**BTC**	${\hat{L}}_{k}P\left(H_{k}\right)$	$P\left(H_{k}\right)$	$\ln\left({\hat{L}}_{k}\right)-k\ln\left(2\right)$	**−4.75**	**−5.30**	**−5.55**
**BIC**	${\hat{L}}_{k}P\left(H_{k}\right)n^{-k/2}$	$P\left(H_{k}\right)$	$\ln\left({\hat{L}}_{k}\right)-k\ln\left(2\right)$	**−5.15**	**−6.11**	**−6.77**
**AIC**	${\hat{L}}_{k}e^{-k}$	−	$\ln\left({\hat{L}}_{k}\right)-k$	**−5.06**	**−5.92**	**−6.47**

## Data Availability

No new data were created or analyzed in this study. Any questions concerning the data used can be directed to the corresponding authors.
